# Production of Belite Based Clinker from Ornamental Stone Processing Sludge and Calcium Carbonate Sludge with Lower CO_2_ Emissions

**DOI:** 10.3390/ma15072352

**Published:** 2022-03-22

**Authors:** Francisco Roger Carneiro Ribeiro, Regina Célia Espinosa Modolo, Marlova Piva Kulakowski, Feliciane Andrade Brehm, Carlos Alberto Mendes Moraes, Victor Miguel Ferreira, Esequiel Fernandes Teixeira Mesquita, Afonso Rangel Garcez de Azevedo, Sergio Neves Monteiro

**Affiliations:** 1 Civil Engineering Graduate Program, Vale do Rio dos Sinos University (UNISINOS), São Leopoldo 93022-750, Brazil; reginaem@unisinos.br (R.C.E.M.); marlovak@unisinos.br (M.P.K.); felicianeb@unisinos.br (F.A.B.); cmoraes@unisinos.br (C.A.M.M.); 2 Mechanical Engineering Graduate Program, Vale do Rio dos Sinos University (UNISINOS), São Leopoldo 93022-750, Brazil; 3 Department of Civil Engineering, University of Aveiro (UA), 3810-193 Aveiro, Portugal; victorf@ua.pt; 4 Laboratory of Rehabilitation and Durability of Constructions (LAREB), Campus Russas, Universidade Federal do Ceará, Russas 62900-000, Brazil; emesquita@ufc.br; 5 Civil Engineering Laboratory (LECIV), State University of Norte Fluminense Darcy Ribeiro (UENF), Av. Alberto Lamego 2000, Rio de Janeiro 28013-602, Brazil; 6 Department of Materials Science, Military Institute of Engineering (IME), Square General Tibúrcio 80, Rio de Janeiro 22290-270, Brazil; snevesmonteiro@gmail.com

**Keywords:** industrial solid waste, ornamental rock processing sludge, calcium carbonate sludge, belitic clinker

## Abstract

Environmental concerns have come to the forefront due to the substantial role of the cement industry in the extraction and expenditure of natural resources. Additionally, industrial processes generate a considerable amount of waste, which is frequently disposed of inadequately. The objective of this study was to evaluate the simultaneous use of ornamental rock processing sludge and calcium carbonate sludge generated from the kraft process in the production of belitic clinker. These waste materials would be used in total or partial substitution of natural raw materials, namely, limestone and clay. Several formulations were produced and sintered at 1100 and 1200 °C. The raw materials were characterized physico-chemically and thermogravimetrically, with subsequent evaluation of the resulting dosed raw mixes. Mineral analyses determined that the mixtures with limestone and clay in substitution ratios of 95% and 100%, respectively, and sintered at 1100 °C have the potential to produce belite-rich clinkers. This temperature is considerably lower than those reported in reference studies. Additionally, full limestone and clay substitution could result in a 23.92% reduction in carbon dioxide in clinker production. The results confirmed the potential use of ornamental rock processing sludge and calcium carbonate sludge as viable alternative materials for cement production and, consequently, could contribute to a reduction in the negative environmental impacts of this industry.

## 1. Introduction

The cement industry consumes a substantial amount of natural resources, generates a high level of carbon dioxide (CO_2_) emissions and, yet, has the potential of recycling industrial solid wastes. Clinker production, in particular, requires an abundant amount of raw materials and consumes fossil fuels that generate elevated levels of greenhouse emissions. Worldwide cement production peaked at 4.2 billion tons in 2015. The estimated production in 2020 was 4.1 billion tons with future projections of 4.7 billion tons by 2050 [1,2,3]. These production figures are highly relevant, since the cement industry was responsible for 8–9% of anthropic CO_2_ emissions [4]. Brazilian emissions are estimated at approximately 866 kg of CO_2_ per ton of clinker and 564 kg of CO_2_ per ton of cement produced.

According to Mikulčić et al. [5], the substitution of non-renewable natural resources with alternative materials could reduce both energy consumption and CO_2_ emissions in clinker production. However, this process must be conducted carefully due to the chemical changes in cement and in order to ensure the final quality of the product. Additionally, a reduction in the availability of calcium oxide (CaO) would result in a binder with a higher level of belite (2CaO·SiO_2_) instead of alite (3CaO·SiO_2_). This change in mineral composition would decrease CO_2_ emissions by 10–15%, thermal energy use by 12–20%, and electrical energy use by 10–15% when compared to ordinary Portland clinker production [6,7,8]. Sui et al. [9] also noted that compared to Portland cement, belitic cements released less heat and had higher workability and strength, increased durability, and less shrinkage from drying over advanced ages.

Studies have indicated that several industrial solid wastes could be successfully used as total replacement for limestone and clay as sources of calcium or silica [10,11,12,13].

Buruberri et al. [10] produced belitic cement at 1390 °C solely from paper industry wastes generated from the kraft process, namely, fly ash, biological sludge, and calcium carbonate sludge. Mortars produced with this cement had a mechanical strength of approximately 13 MPa after 90 days, which is suitable for use in external and internal coatings. Additionally, there were no durability issues from efflorescence or other potentially damaging effects.

Ávalos-Rendón et al. [11] sinterized two reactive belitic cements from active silica and commercially available natural zeolite at a temperature of 1000 °C. The results showed better mechanical strength than Portland cement at initial ages and above 14 days. Improved hydraulic reactivity was also observed particularly with active silica belitic cement with a hydration degree of approximately 33% at 28 days compared to 26% for Portland cement.

Vashistha et al. [12] sinterized belitic clinker based exclusively on calcium carbonate sludge, nano-silica, and furnace ash. Belite was produced from the combined action of calcium carbonate sludge and nano-silica at 1000 °C without pre-calcination or chemical stabilizers. In comparison, belite was produced from a mixture of calcium carbonate sludge and furnace ash between 1100 and 1200 °C. Cement with calcium carbonate sludge and nano-silica were observed to have a compression strength of approximately 13 MPa at 56 days, which is suitable for internal and external coatings. Additionally, there was a measurable reduction in the hydration heat.

Enríquez et al. [13] evaluated belitic clinker production from carbonate calcium sludge, cement furnace ash, and rice husk ash without the use of limestone and clay. Belitic-based clinker was obtained at 1350 °C with high hydration kinetics and large heat release at initial ages.

In this context, besides the pulp and paper industry, the ornamental stone industry generates large amounts of waste. In this study, we considered the calcium carbonate sludge and the sludge from the processing of ornamental rocks. These types of industrial waste are frequently disposed in unregulated sanitary landfills, waterways, or shorelines and become a great source of air, water, and soil contamination [14,15]. A circular economy in these spaces offers a way to minimize the environmental and economic costs of waste with a strategic focus on reformulating materials and energy flow to achieve greater resource efficiency through reuse, remanufacturing, and recycling [16].

Ornamental rock production in Brazil amounted to 9 million tons in 2020 [17]. Ornamental rock processing sludge (ORPS) starts with the cutting and extraction of rectangular blocks of solid rock material of varying sizes and volumes between 8 and 15 m^3^. The rectangular shape allows easier processing into slabs with thicknesses between 2 and 3 cm and surfaces polished to a glassy appearance. These steps make use of a substantial amount of water for cooling, cutting, and polishing. The result is a conversion of approximately 58% of the total volume of the block into sludge, which represents a considerable waste of natural resources [18,19,20]. As noted by Karaca et al. [21], the exact amount of waste generated depends on the geological, textural, and petrographic characteristics of the rock, type of cutting machines, plant processing capacity, and block size.

The cellulose and paper industries have a considerable environmental impact. Wood-based production generates a large amount of waste materials, liquid effluent, and atmospheric pollution due to the emission of carbon dioxide, methane, and nitrous oxide [22,23]. The generated waste contains organic material and toxic substances from the additives and chemical products used in the manufacturing process, which requires specialized handling [24]. The amount of each type of waste depends on the amount of unprocessed raw material and the type of paper to be produced [25]. It was estimated that 0.2–0.3 kg of calcium carbonate sludge (CCS) was generated for each ton of processed cellulose in Brazil.

Thus, the purpose of this study was to investigate the potential use of industrial wastes, specifically ORPS and CCS, for clinker production. The results were of particular innovative importance since, at the moment, there are no reference works that have evaluated the combined use of ORPS and CCS. While not all chemical, physical, mineralogical, and thermal results on clinker/cement formulations were discussed in-depth, this study presents relevant information for the understanding of belitic cement production from substitute materials. Additionally, while large-scale cement production has specific procedures related to raw material composition, operation, and equipment, laboratory-scale results could still provide preliminary insight into the ideal mix ratios and other parameters that affect phase formation and cement performance.

## 2. Materials and Methods

### 2.1. Materials

The materials used in this study were limestone, kaolinite clay, ornamental rock processing sludge (ORPS) from slab polishing, and calcium carbonate sludge (CCS) from the kraft process of cellulose and paper. The limestone and clay were ground in a ball mill until particle sizes of less than 75 μm were reached. Two different shipments of each type of waste were received and then homogenized to obtain a meaningful composition. All materials were previously dried in an oven at 100 ± 5 °C for 24 h. Chemical characterization was conducted with X-ray fluorescence (XRF), and the results were used to determine the ratios of the raw mixtures.

### 2.2. Methods

#### 2.2.1. Dosage and Production of Belitic Clinker

Sample mix ratios were calculated and refined with the lime saturation factor (LSF), silica modulus (SM), alumina modulus (AM), and Bogue [26] formulas. These are shown in Equations (1)–(7) based on the elemental chemical composition of the materials and constraints of the desired mineral content. The mix ratios were formulated in order to obtain the main phases of the clinker and to maximize industrial waste reuse.

(1) $$\mathrm{LSF}=\;\frac{\mathrm{CaO}}{2.80\;{\mathrm{SiO}}_{2}+1.20\;{\mathrm{Al}}_{2}O_{3}+0.65\;{\mathrm{Fe}}_{2}O_{3}}$$

(2) $$\mathrm{SM}=\frac{{\mathrm{SiO}}_{2}}{{\mathrm{Al}}_{2}O_{3}+{\mathrm{Fe}}_{2}O_{3}}$$

(3) $$\mathrm{AM}=\frac{{\mathrm{Al}}_{2}O_{3}}{{\mathrm{Fe}}_{2}O_{3}}$$

(4) $$C_{3}S=(4.071\;\times \;\%\mathrm{CaO})\;–\;(7.600\;\times \;\%{\mathrm{SiO}}_{2})\;–\;(6.718\;\times \;\%{\mathrm{Al}}_{2}O_{3})\;–\;(1.430\;\times \;\%{\mathrm{Fe}}_{2}O_{3})\;–\;(2.852\;\times \;\%{\mathrm{SO}}_{3})$$

(5) $$C_{2}S=(-3.071\;\times \;\%\mathrm{CaO})+(8.600\;\times \;\%{\mathrm{SiO}}_{2})+(5.068\;\times \;\%{\mathrm{Al}}_{2}O_{3})+(1.079\;\times \;\%{\mathrm{Fe}}_{2}O_{3})+(2.152\;\times \;\%{\mathrm{SO}}_{3})$$

(6) $$C_{3}A=(2.650\;\times \;\%{\mathrm{Al}}_{2}O_{3})\;–\;(1.692\;\times \;\%{\mathrm{Fe}}_{2}O_{3})$$

(7) $$C_{4}\mathrm{AF}=(3.043\;\times \;\%{\mathrm{Fe}}_{2}O_{3})$$

After characterization of the source materials, four formulations were designated for clinker production. These are shown in Table 1 in w.t.%. The REF reference sample contained no wastes, while formulations F1, F2, and F3 contained only wastes in different proportions and no limestone or clay. Table 1 also shows the values of the chemical modules used in the mixing ratios and the phase contents predicted from the Bogue calculation.

The industrial wastes of this study were considered alternate sources of the main chemical elements in clinkers: SiO_2_, Al_2_O_3_, CaO, and Fe_2_O_3_. As such, they allowed for elevated reductions in the use of natural raw materials.

Regarding the mix ratios of natural materials and wastes, decreases in limestone saturation factor (LSF) were known to increase belite content and with this factor, the flours were dosed and produced with LSF ranging from 70% to 80% and CaO/SiO_2_ ratios ranging from 2.46 to 3.00. The SM and AM module were also within the range of traditional cements, which ensured an ideal distribution between calcium silicates and aluminate phases [27].

According to Stark et al. [28], a reduction in the lime saturation limit leads to a higher belite content and a lower alite content. Bouzidi et al. [29] stated that a completely belitic clinker could be produced with a CaO/SiO_2_ ratio of 2. However, Clarizka et al. [30] reported intense belite peaks with a CaO/SiO_2_ ratio of 3, which decreased as the ratio decreased. 

Table 1 also showed that for formulations F1, F2, and F3, as the substitution level of both wastes decreased and limestone use increased, silica and alumina moduli tended to decrease. This was a result of limestone containing lower silica and alumina moduli in its chemical composition when compared with ORPS but not with CCS. Additionally, C_3_S levels increased while C_2_S and C_3_A levels decreased in the Bogue calculation estimates due to the increase in impurities associated with limestone. However, formulation F3 with more limestone had an increase in C_4_AF. This was a consequence of the lower Al_2_O_3_/Fe_2_O_3_ content of limestone when compared to clay and ORPS. These results confirmed the cited phenomenon of lower belite content as limestone content increased.

Taylor [31] reported that for Portland cement clinker, an increased silica modulus decreased the proportion of liquid phases at any oven temperature and increased the complexity of the clinkering process. Additionally, decreasing the alumina modulus increased the levels of iron compounds, which acted as fluxes and favored clinkering at lower temperatures.

To produce the clinkers, the dried materials were homogenized in a cylindrical grinder at 60 rpm for 1 h and transferred to a clean receptacle. The grinder was thoroughly cleaned between operations. A total of two reference formulations and six waste-only formulations were prepared at 20% moisture to be subjected to different sintering temperatures.

**Figure 1:**
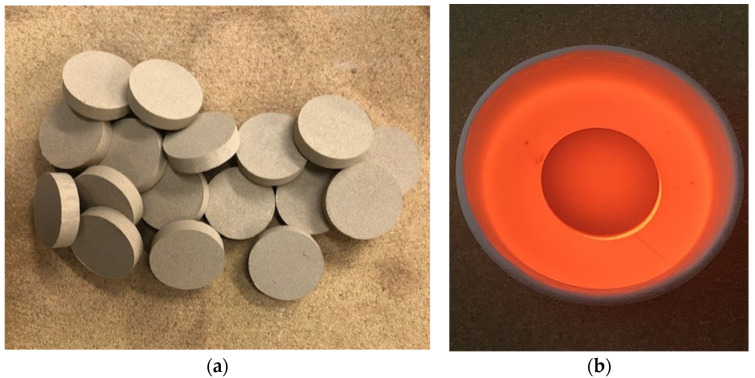
Tablet samples: (**a**) after pressing and (**b**) after clinkering.

Samples were shaped as round tablets with 10 g of dry mixture and 2 g of deionized water as seen in Figure 1a. Tablets were pressed in a manual hydraulic press with 30 tons for 10 min, followed by thermal treatment. Tablets were placed in an alumina crucible and subjected to a pre-calcination treatment at 900 °C and clinkering at either 1100 °C or 1200 °C in a high-temperature electrical oven as seen in Figure 1b. Details of the control conditions of the thermal cycles are shown in Table 2.

After thermal cycling, all clinker samples were forcibly cooled under air convection in order to prevent polymorphic transformations of the calcium silicates and accentuated crystallization of liquid phases. Cooling also ensured a prevalence of dicalcium silicate (β-C_2_S). Since the samples had elevated hardness, they were first fragmented in a roller crusher and then ground in an eccentric ball mill grinder with alumina balls in order to obtain particles smaller than 63 μm. Particles were subjected to mineral and physical characterization, and it should be noted that no gypsum was added during clinker production.

#### 2.2.2. Material Characterization

Physical characterization was conducted through laser granulometry in a Microtrac apparatus, model S3500, Osaka, Japan. This technique was based on the relationship between particle sedimentation and light absorption to determine the granulometric distribution. In the case of particle agglomeration from humidity, sodium hexametaphosphate was used to break down clusters, and water was used as a solvent. Specific mass was determined from helium gas pycnometry with a Micromeritics apparatus, model AccuPyc II 1340, Norcross, EUA, as helium easily penetrated the pores of the samples. Specific surface area was determined from nitrogen adsorption using the BET method with a Micromeritics apparatus, model TriStar II Plus, Norcross, EUA. Larger specific surface areas were indicative of dimensions and, by extension, higher reactivity of the particles.

Chemical characterization was performed with energy-dispersive X-ray fluorescence (XRF) using a PANalytical apparatus, model Epsilon 1, São Paulo, Brazil. Prior to characterization, samples with a mass between 1 and 10 g were dried in an oven at 105 ± 5 °C. The results identified chemical composition in terms of major and minor oxide quantities.

Thermal analyses by thermogravimetry (TG) and differential thermogravimetry (DTG) were performed with a PerkinElmer simultaneous thermal analyzer, model STA 8000. Each test made use of approximately 20 mg of material in an alumina crucible heated at a rate of 10 °C/min in a N_2_ environment in a temperature range from 25 to 1000 °C. The results allowed for the evaluation of mass gain or loss under continuous or uniform heating and thermal decomposition reaction kinetics.

Morphological and microstructural characterizations of the wastes were conducted through scanning electronic microscopy (SEM) in a model EVO MA 15 apparatus, Urbana, EUA. Samples were metalized in carbon and analyzed in a vacuum with a secondary electron detector operating at 20 kV.

Mineral analysis of the wastes and clinkers produced were conducted through X-ray diffraction (XRD) in a PANalytical apparatus, model Empyrean, São Paulo, Brazil. The analyzer contained a copper source and operated with a tension of 40 kV and current of 40 mA. Analysis made use of an angle variation between 5° and 70°, a step of 0.0131, and a time of 97.92 s for each step. The identification of the crystalline phases was made using the X’Pert HighScore Plus software.

#### 2.2.3. Evaluation of Carbon Dioxide Emissions during Clinker Production

Thermal analyses were performed to confirm CO_2_ emission estimates per ton of each formulation from the sintering process. This was based on the method proposed by Costa and Ribeiro [32] and consisted of TG and DTG on the raw materials and formulations. The analysis temperature varied from 550 to 850 °C, which was the main interval of mass loss from decarbonation. Residual masses were taken at 1000 °C, and contributions from the expenditure of thermal and electrical energies were not considered.

## 3. Results and Discussion

### 3.1. Characterization of Raw Materials

**Figure 2:**
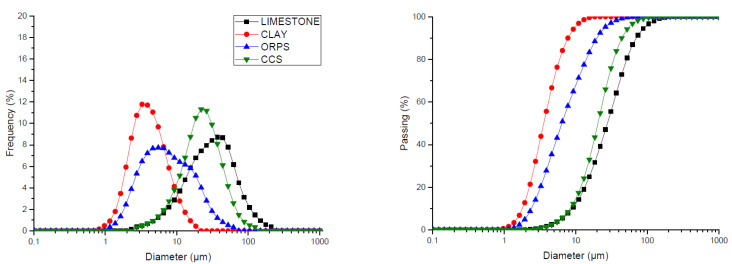
Granulometric distribution of the raw materials.

The chemical and physical compositions of the raw materials and wastes used in this study are shown in Table 3 with respect to the more stable oxides. The granulometric distribution of the raw materials used in this study are shown in Figure 2.

The results in Table 3 demonstrate that the chemical composition of the wastes was directly related to their source and treatment during the production process. The selected solid industrial wastes were confirmed as potential substitutes for the natural raw materials used in clinker production, namely, ORPS could replace clay to supply silica and alumina, while CCS could replace limestone to supply calcium oxide.

Limestone contained a 43.84% d.b. prevalent content of calcium oxide (CaO) and 12.59% d.b. of quartz (SiO_2_). Clay was of kaolinitic type with aluminum silicate characteristics. Characterization determined a 64.40% d.b. prevalent content of quartz (SiO_2_) and 19.86% d.b. of alumina (Al_2_O_3_). In comparison, ORPS contained quartz, calcite, and alumina, which differed from the chemical compositions described by Bacarji et al. [33], Sato et al. [34], and Awad et al. [35]. No pozzolanic activity was noted in ORPS, but despite being inert with respect to reactivity, its MgO, Na_2_O, and K_2_O content were expansive oxides which have effects that must be considered in the production of cementitious compounds. Similar to limestone, CCS also presented a prevalent 55.49% d.b. content of calcium oxide and 42.51% of loss of ignition. One of the advantages of using this waste is that it does not require grinding and requires much less energy to prepare raw clinker.

The elevated levels of calcium oxide both in limestone and CCS were related to the common presence of calcium carbonate (CaCO_3_) in both materials. Since the measured reduction in mass from the loss of ignition was the result of the release of CO_2_ from calcium carbonate decomposition, it was possible to estimate the initial amount of calcium carbonate from a stoichiometric calculation. Based on the mass fraction of CaO from Table 3, a 78.29% fraction of calcium carbonate in the limestone was determined, and the amount of calcium carbonate in CCS was estimated with a stoichiometric calculation of 99.09%. This high purity CCS indicates the need for use in smaller quantities to replace limestone.

Furthermore, the results of Table 3 show that limestone and clay particle diameters were measured in ranges of 1–125 μm and 1–22 μm, respectively. In comparison, ORPS and CCS particle diameters ranged between 1 and 62 μm and 2–105 μm, respectively. Since all particles had diameters of less than 75 μm, they were considered suitable for homogenization and clinker production. The smaller particles had increased surface areas that favored clinkering reactions. 

**Figure 3:**
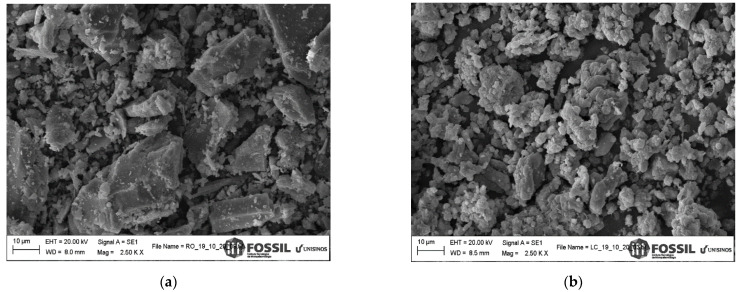
Industrial waste morphology: (**a**) ORPS and (**b**) CCS.

Industrial waste morphologies are shown in the images in Figure 3. The surface texture of ORPS was rough with irregular, angular, and plaque-shaped particles due to the slicing, molding, and polishing procedures. In comparison, the CCS texture consisted of irregular and bunched particles, probably due to the precipitation and settling production processes.

**Figure 4:**
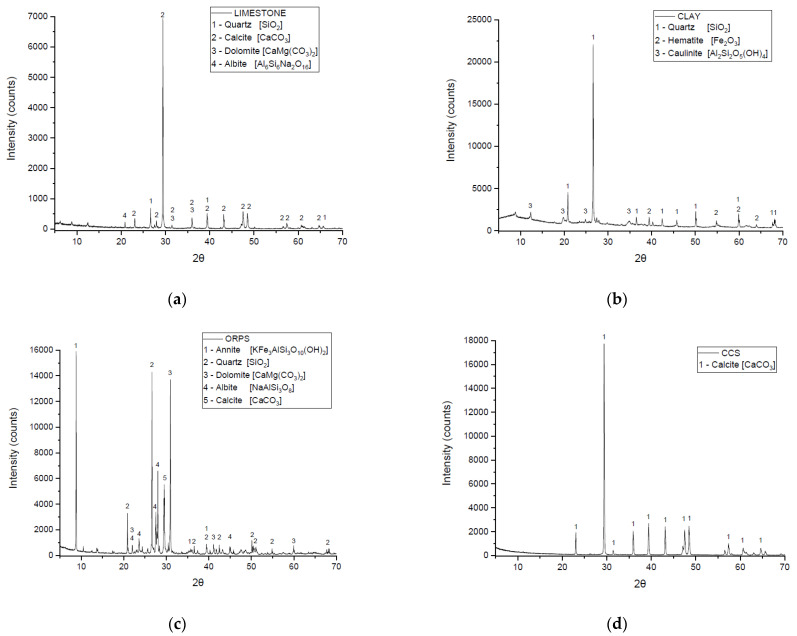
Mineral characterization of the raw materials. Limestone (**a**), Clay (**b**), ORPS (**c**), and CCS (**d**).

Figure 4 presents the mineral characteristics of the materials used in this study through individual diffractograms. As expected, the results agree with the chemical composition presented in Table 3. 

The limestone diffractogram in Figure 4a identified mainly crystalline peaks of quartz and calcite. According to Abrahão [36], silica in limestone was common and could be represented by microcrystalline quartz. The clay diffractogram in Figure 4b identified quartz, hematite, and kaolinite phases. The ORPS diffractogram in Figure 4c presented mainly peaks of quartz and calcite and minor peaks of annite and albite in agreement with Mesquita, Dall’Agnol, and Almeida [37] and Lima et al. [38]. Minor peaks of silicate minerals from the mica and feldspar groups were also found in ORPS. Lastly, the diffractogram of CCS in Figure 4d identified mostly calcite peaks.

### 3.2. Mineral Characterization of Clinker Formulations

**Figure 5:**
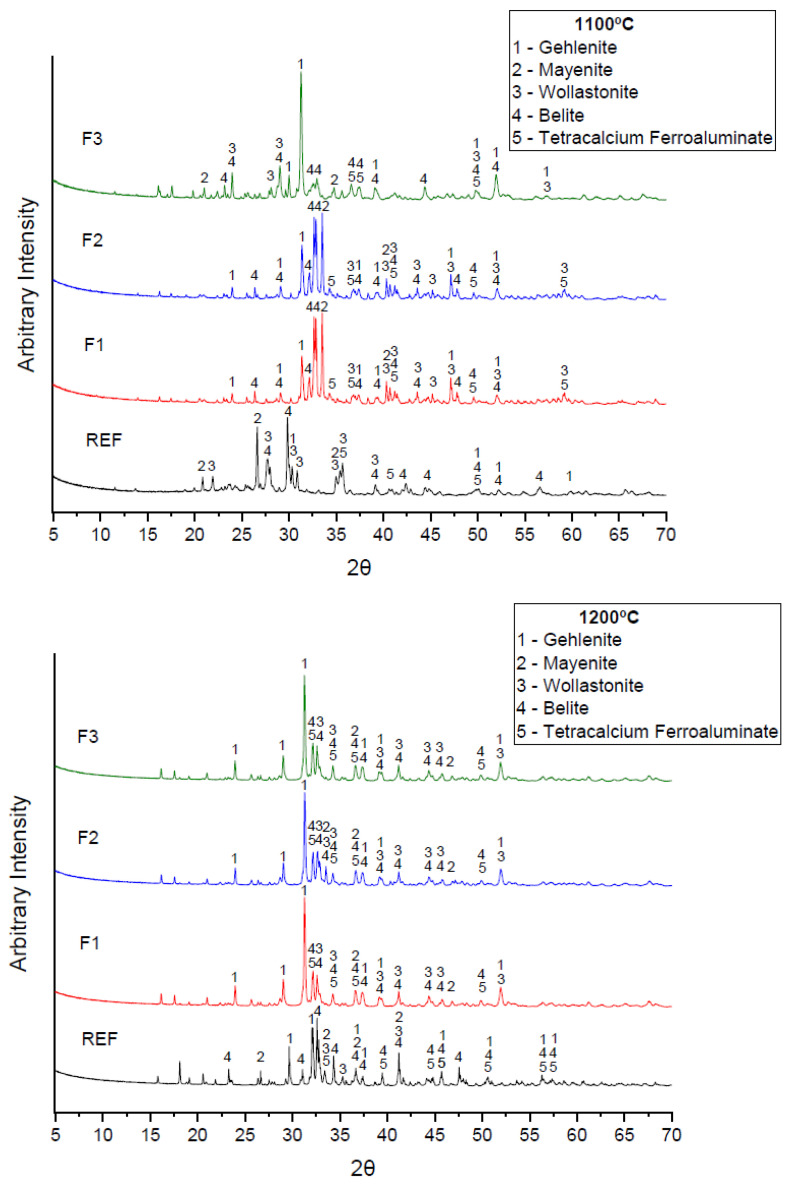
Mineral analysis of experimental clinkers produced.

Figure 5 presents the diffractograms of all formulations in this study at sintering temperatures of 1100 and 1200 °C. All diffractograms contained crystalline peaks of gehlenite (Ca_2_Al_2_SiO_7_), mayenite (Ca_12_Al_14_O_33_), wollastonite-1A (CaSiO_3_), belite (β-Ca_2_SiO_4_), and tetracalcium aluminoferrite (Ca_4_Al_2_Fe_2_O_10_).

Despite rigid control under laboratory conditions, there were difficulties in replicating the exact same conditions for each batch. Consequently, small differences in mineral content were possible. The absence of alite in the diffractograms may have resulted from an instability in the cooling process, reverting to the more stable dicalcium silicate [39] or by the presence of potassium present in the sludge from the processing of ornamental rocks that hindered the reaction of belite with CaO [13].

Formulations F1 and F2 sintered at 1100 °C presented dicalcium silicate (β-C_2_S) as the majority phase. This phase crystallized at high temperatures and, similar to α-polymorph, was highly reactive to cement according to Kacimi et al. [40] and Mindat [41]. Its presence could be related to efficient rapid cooling, which was reported by Kacimi et al. [40] and Bouzudi et al. [29] as a pre-requisite for the production of reactive belitic cements. Belite could also be stabilized through doping with substances such as boron, phosphorus, sulfur, sodium, and potassium as recommended by Elfami et al. [42] and Saidani et al. [43].

Under different temperatures, the results of formulations F1 and F2 detected expressive belite peaks sintered at 1100 °C and lesser peaks at 1200 °C. The increase in crystallization of this phase could be related to the size of the silica nanoparticles after the clinkers were ground. A combination of increased specific surface area and lower particle dimension induced chemical reactions at lower temperatures with a higher reaction rate due to the electrostatic attractive forces. Maheswaran et al. [44] also observed higher belite peaks at lower in clinkering temperature for formulations with calcined lime sludge and silica fume.

Formulations REF and F3 at 1100 °C and formulations F1, F2, and F3 at 1200 °C presented gehlenite as the majority phase. Gehlenite is a calcium aluminosilicate with low hydraulic properties. Its formation was related to elevated silica content [45] such as the case with ORPS or elevated alumina content as per Zhou et al. [46]. Limited quantities of gehlenite were considered tolerable [47], and its formation was noted both under laboratory and industrial clinker production [48]. However, such formulations should not be considered as having characteristics of belitic clinkers. Han et al. [49] noted that gehlenite could act as an inert material to increase the structural density of the clinker. However, Doval et al. [50] and Winnefeld et al. [51] cautioned that small particles of gehlenite were more reactive and could form strätlingite over long time periods, even more if impurities such as K^+^ or Na^+^ were present as they increased reactivity [52].

No free lime or periclase was observed in any formulation considering the decrease of LSF to values close to 75%. Hydrated excess free lime can form calcium hydroxide (Ca(OH)_2_) which, due to the fact of its expansion characteristics, may result in negative effects on mechanical strength and durability of cementitious materials [31]. 

Mayenite was detected in place of tricalcium aluminate, and its presence could have been the result of low LSF, low alumina content in the raw materials, and the combination of lime and silica. It is considered to be an easily hydrated phase with slightly less reactivity than tricalcium aluminate (C_3_A). It is important in cements with high initial strength but in carefully controlled quantities [53,54]. Carvalho et al. [55] noted that mayenite is acceptable in belitic cements, since it could result in the same increase in the initial mechanical strength.

Wollastonite formation was a consequence of a SiO_2_ and CaCO_3_ reaction. This was confirmed as ORPS and CCS increased the silica and calcium content, respectively, in substitution of limestone and clay. Wollastonite formation from β-C_2_S and leftover silica in the raw materials was also responsible for the considerable decrease in belite peak intensity at the higher temperature of 1200 °C as explained in the study by El-Didamony et al. [56]. As reported by Qu et al. [57], a balance favoring β-C_2_S over wollastonite is related to a Ca/Si ratio of 3. For ordinary Portland cement (OPC), a Ca/Si ratio of 3.2 resulted in belite and lime, while a ratio lower than 2 resulted in gehlenite and wollastonite at 800 °C, as the alkali content lowered the crystallization temperature. Consequently, for this study, Ca/Si ratios were set at approximately 2.5 in order to favor belite formation and stabilization with respect to other phases.

The tetracalcium aluminoferrite (Ca_4_Al_2_Fe_2_O_10_) phase was identified in orthorhombic form (Pcmn) as brownmillerite. It was formed at temperatures of 1100 °C from the cooling of a Fe_2_O_3_-rich liquid phase according to Garcia-Mate et al. [58]. It should be noted that Fe_2_O_3_ was one of the main oxides both in clay and ORPS. The hydraulic capacity of this phase depended on the production procedures, and its reactivity increased up to temperatures of 1200 °C [8].

Both Gartner and Sui [7] and Kotsay and Jaskulski [59] stated that sintering temperatures of belite-rich cements could vary between 1000 and 1350 °C. In formulations F1 and F2 in this study, higher intensity belite peaks were identified at a temperature of 1100 °C, which was lower than expected from reference works, and it was produced with waste only.

### 3.3. Clinker Particle Sizes

Table 4 presents the granulometric distribution and specific surface area from BET of the belitic clinkers of this study in accordance with sintering temperature.

The results in Table 4 denote that grounding process was similar for all formulations, as it resulted in minimal variations in diameter. Another contributing factor to the uniformity of the results would be the similar mineral composition of the clinkers. 

The specific surface area for these cements was determined so that it relates to the hydraulic reactivity that may occur. Smaller specific surface area values were found for formulations F1 and F2 at 1100 °C, which could be the result of higher reactivity due to the presence of β-C_2_S. The remaining higher specific surface areas could be assumed to be the effect of the presence of gehlenite and/or porosity of the particles. These results are similar to those found in the research of Kacimi et al. [40] and Koumpouri and Angelopoulos [60].

### 3.4. Evaluation of Carbon Dioxide Emissions during Clinker Production

**Figure 6:**
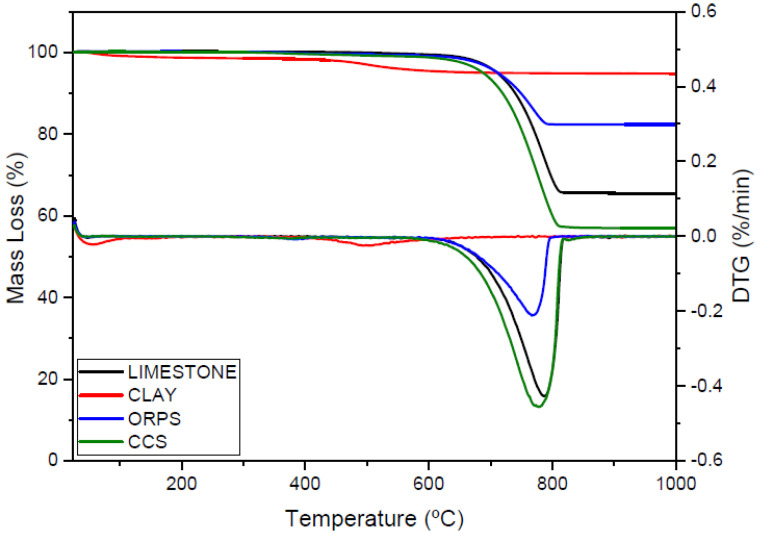
Thermogravimetric analyses of the raw materials.

Thermogravimetric analyses of limestone, clay, ORPS, and CCS in natura are shown in Figure 6. The total mass losses of limestone and calcium carbonate sludge were 33.66% up to 787.85 °C and 41.72% up to 778.32 °C, respectively. These peaks were characteristic of calcium carbonate decomposition. The mass loss observed for ORPS was 16.67% up to 768.69 °C and was also related to the decarbonation of small amounts of calcite. The clay showed a mass loss of 3.74% up to 501.23 °C and was related to the dehydroxylation of kaolinite. The mass losses at the beginning of the heating process were related to water release and the burning of organic matter. The cited mass losses agreed with the chemical characterization results.

**Figure 7:**
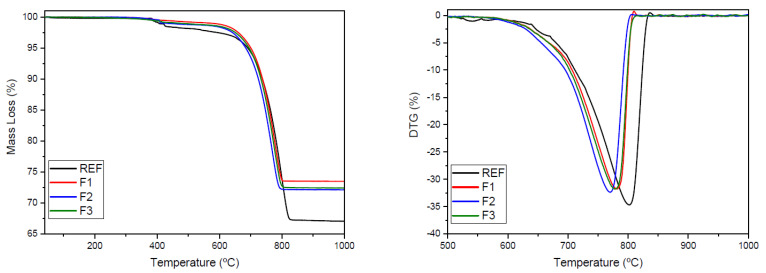
Thermogravimetric analysis of the raw clinker mixtures.

Figure 7 presents thermogravimetric curves of the raw clinker mixtures of this study. Based on mass loss, it was possible to estimate the CO_2_ emissions per ton of clinker produced, as shown in Table 5.

As seen in Table 5, the REF formulation, with 90% limestone and 10% clay, had the highest CO_2_ generation (decarbonation) measured at 30.68%. This result was expected due to the high lime content of the formulation. For the clinkers, formulation F1 containing only ORPS and CCS presented the least amount of CO_2_ release at 25.58%, followed by formulations F2 and F3 with 26.33% and 25.58%, respectively. These values did not take into account emissions from thermal and electrical energy expenditures. Theoretically, the use of ORPS and CCS increased CaO levels from dissociated CO_2_, and the mix ratios used were ideally suited to minimizing CO_2_ emissions. Consequently, these industrial wastes could have a direct impact both on reducing emissions and demand on natural resources.

Table 5 also demonstrates that all waste-containing clinker formulations in this study presented lower CO_2_ emissions than the REF formulation. The full industrial waste substitution of formulation F1 resulted in 255.80 kg of CO_2_ generated per ton of raw mixture and 347.98 kg of CO_2_ generated per ton of clinker produced. In terms of reductions in emissions, the substitution formulations presented decreased CO_2_ between 19.83% and 23.92%, which are significant values for the cement industry. These considerable reductions were due to the lower use of lime in the mixtures, since the wastes were able to supply the same essential substances, such as calcium oxide, to the process [61].

## 4. Conclusions

The combined use of ornamental rock processing sludge (ORPS) and calcium carbonate sludge (CCS) as substitutes for natural raw materials could be considered as viable for the production of belitic clinkers. The main conclusions of this research were:

- Both ORPS and CCS presented chemical and physical characteristics that favored clinkering;
- Mineral characterization identified predominantly belite in its meta-stable state (β-C_2_S), with prevalent crystalline peaks in formulations F1 and F2 at 1100 °C. The CaO/SiO_2_ ratio of the formulations tested in this study were within the pre-requisites for the production of belitic cements;
- The absence of a γ-C_2_S phase in the diffractograms attested to the efficiency of rapid clinker cooling, which prevented undesirable polymorph formation;
- Formulations REF and F3 formed gehlenite in both sintering temperatures, which was undesirable due to the fact of its lack of hydraulic properties;
- Clinker granulometry results were similar for all formulations and was related to the mineral characteristics;
- Formulations F1 and F2 presented lower CO_2_ emissions per ton of clinker produced. Formulations with 95–100% replacement by solid industrial waste could be used to produce belitic clinkers;
- Partial reduction or total substitution of limestone and clay could bring environmental and economic benefits to both cement and waste-generating industries.

It should be noted that the substitution ratios used in this research were not viable for widespread application due to the relatively low volume of waste generated from ornamental rock and paper industries compared to the elevated demand of raw materials in the production of cement. However, bench scale production of belitic cements with industrial waste, exclusively, could be an important strategy in the use of alternative materials.

Nonetheless, the combined use of ORPS and CCS could result in reductions in CO_2_ emissions and lower energy consumption in the production of belitic cements. The results of this research represented a forward step in the preservation of non-renewable natural resources, minimization of improper disposal of solid industrial wastes, and the search for more sustainable and cleaner production.

## Figures and Tables

**Table 1:** Experimental clinkers and mix ratios.

Source Materials, Chemical Moduli, and Theoretical Phase Content	Formulations and Respective Mix Ratios (w.t.%)			
	REF	F1	F2	F3
Limestone	90.0	0.00	5.00	10.0
Clay	10.0	0.00	0.00	0.00
ORPS	0.00	52.5	50.0	47.5
CCS	0.00	47.5	45.0	42.5
LSF	77.44	77.64	77.92	79.50
SM	2.46	3.00	2.97	2.94
AM	2.49	2.66	2.63	2.60
C_3_S	11.44	15.68	18.55	21.42
C_2_S	64.80	64.01	61.15	58.30
C_3_A	14.67	12.91	12.82	12.74
C_4_AF	9.08	7.33	7.39	7.46

**Table 2:** Details of the pre-calcination and clinkering thermal cycles.

Cycle A	Cycle B	Cycle C	Cycle D	Cycle E
900 °C HR = 5 °C/min	900 °C HT = 30 min	1100 °C HR = 10 °C/min	1100 °C HT = 60 min	Forced convection air cooling
		1200 °C HR = 10 °C/min	1200 °C HT = 60 min	

HR = heating rate; HT = heating time.

**Table 3:** Chemical composition and physical characterization of the raw materials.

Chemical Characterization (%)		Limestone	Clay	ORPS	CCS
SiO_2_		12.59	64.40	36.89	ND
Al_2_O_3_		3.57	19.86	8.51	0.36
Fe_2_O_3_		1.69	4.62	3.31	0.04
CaO		43.84	ND	24.50	55.49
MgO		1.08	1.24	5.13	0.71
SO_3_		ND	ND	0.01	0.05
Na_2_O		0.28	0.20	1.62	0.56
K_2_O		0.63	4.36	3.16	0.01
SrO		0.12	0.03	0.06	0.25
MnO		0.06	0.05	0.05	0.01
P_2_O_5_		0.12	0.46	0.09	ND
TiO_2_		0.24	0.65	0.81	0.01
Loss of Ignition (LOI)		35.78	4.13	15.86	42.51
Specific Surface Area BET (cm^2^/g)		33,491	26,687	26,819	12,566
Specific Mass (g/cm^3^)		2.65	2.60	2.66	2.59
Granulometric Analysis	D_10_ (μm)	8.96	1.81	2.42	8.41
	D_50_ (μm)	27.82	3.58	6.41	20.47
	D_90_ (μm)	69.84	7.78	19.66	43.96
	D_M_ (μm)	32.22	4.06	8.40	22.70

**Table 4:** Physical characteristics of experimental clinkers.

Formulations		Granulometric Analysis				Specific Surface Area BET (cm^2^/g)
		D_10_ (μm)	D_50_ (μm)	D_90_ (μm)	Dm (μm)	
**1100 °C**	**REF**	1.83	4.59	10.51	5.27	4.418
	**F1**	1.34	3.83	9.47	4.51	4.052
	**F2**	1.51	3.98	9.12	4.56	4.076
	**F3**	1.25	2.89	6.44	3.30	4.787
**1200 °C**	**REF**	1.27	2.91	7.00	3.42	4.517
	**F1**	1.60	3.60	8.47	4.22	4.629
	**F2**	1.59	3.17	6.59	3.57	4.821
	**F3**	1.63	3.32	6.96	3.74	4.913

**Table 5:** Thermogravimetric analysis of the raw clinker mixtures.

Formulation	Mass Loss from Decarbonation (%)	kg of CO_2_/ton of Clinker	Residual Mass at 1000 °C (%)	CO_2_ Emission (kg)/ton of Raw Mixture (t)	CO_2_ Emission (kg)/ton of Clinker (t)	Reduction in CO_2_/ton of Raw Mixture (%)	Reduction in CO_2_/ton of Raw Mixture (%)
**REF**	30.68	1000	67.08	306.80	457.36	-	-
**F1**	25.58		73.51	255.80	347.98	16.62	23.92
**F2**	26.33		72.15	263.30	364.93	14.18	20.21
**F3**	26.56		72.44	265.60	366.65	13.43	19.83

## Data Availability

Data sharing not applicable.
